# Multiscale Price Lead-Lag Relationship between Steel Materials and Industry Chain Products Based on Network Analysis

**DOI:** 10.3390/e24070865

**Published:** 2022-06-23

**Authors:** Sui Guo, Ze Wang, Xing Zhou, Yanan Wang

**Affiliations:** 1 School of Business, Jiangsu Normal University, Xuzhou 221116, China; dengq@cumt.edu.cn; 2 International Academic Center of Complex Systems, Beijing Normal University at Zhuhai, Zhuhai 519087, China; zach_wang@bnu.edu.cn; 3 School of Systems Science, Beijing Normal University, Beijing 100875, China; 4 Financial Office, Jiangsu Normal University, Xuzhou 221116, China; wangyanan@jsnu.edu.cn

**Keywords:** price lead-lag relationship, steel industry chain, multiscale analysis, cross-correlation method, complex network

## Abstract

As two main steelmaking materials, iron ore and scrap steel have different price lead-lag relationships (PLRs) on midstream and downstream steel products in China. The relationships also differ as the time scale varies. In this study, we compare the price influences of two important steel materials on midstream and downstream steel products at different time scales. First, we utilize the maximal overlap discrete wavelet transform (MODWT) method to decompose the original steel materials and products price series into short-term, midterm, and long-term time scale series. Then, we introduce the cross-correlation and Podobnik test method to calculate and test the price lead-lag relationships (PLRs) between two steel materials and 16 steel products. Finally, we construct 12 price lead-lag relationship networks and choose network indicators to present the price influence of the two materials at different time scales. We find that first, most scrap steel and steel products prices fluctuate at the same time lag order, while iron ore leads most steel products price for one day. Second, products that exist in the downstream industry chain usually lead to iron ore. Third, as the time scale becomes longer, the lead relationships from steel materials to steel products become closer.

## 1. Introduction

The steel industry chain can be divided into the upstream stage (which provides raw materials) and the midstream and downstream stages (which produce steel products) [1,2]. Price variation in upstream raw materials influences not only supply and demand toward themselves but also midstream and downstream steel products. There also exists time scale heterogeneity among the steel industry chain price lead-lag relationships (PLRs). In short-term, midterm and long-term periods, PLRs between materials and products may present different features.

In the steel upstream chain, steel-making industries face trade-offs in choosing raw materials between iron ore and scrap steel. Choosing different materials means taking different production techniques and facing different environmental pressures and costs [3]. Moreover, the PLRs between materials and end-use steel products also differ. Different steel materials cause various downstream steel product prices [4,5]. Another phenomenon that has been considered is the time heterogeneity in PLRs between steel materials and products. In the real market, PLRs between materials and steel products change over time, and the bivariate price influence relationship represents a multiscale phenomenon [6]. Researchers study this time heterogeneity by testing the structural breakpoint and dividing the sample data period into different periods [4]. Additionally, there exists a study that uses the rolling window method to test the time-varying relationship between the iron ore index, scrap steel price, and steel prices [5]. Those studies reveal time-varying price relations by dividing the price series data into different periods, thereby changing the data integrity, and ignore the multi-time-scale information implied in the original price series. These original price time series data contain different time-frequency domain information which is concerned with different types of market entities [7]. Moreover, in the above studies, researchers ignore many steel product prices in the whole industry chain. These researchers use only crude steel prices and steel stock prices to represent the overall steel prices in China.

This study focuses on the time-varying price lead-lag relationships from two iron ore and scrap steel to steel industry chain products in China. By introducing the maximal overlap discrete wavelet transform (MODWT) method, [8], we divide the original data series into six time-scale series, including short-term, midterm, and long-term time scale data. By utilizing the cross-correlation [9] method and the Podobnik test [10], we calculate and filter the PLRs between steel materials and whole industry chain products at different time scales. Finally, by using the complex network method, this study establishes 12 PLR networks to analyze the overall relationship and structural influence between steel materials and industry chain products. The complex network analysis method is a systematic study method which focuses on complicated interaction relationships between elements or individuals in a system. Based on the complex network method, this study can address steel product price interactions in the whole industry chain at the same time. The complex network method is originated from Watts and Strogatz [11], who study the nature of “small-world” of a network, and Barabasi and Albert [12], who study the scale-free nature of network. Then more and more researchers focus on this method and introduce this method into other fields, such as biology network [13], transportation network [14,15], finance network [16,17], and trade network [18,19]. Through abstracting relationships between entities in the real world to nodes and edges of a network, this method can analyze the topological features and the importance of nodes and edges in the network, thereby reflecting the nature and influence of entities in the real-world system.

This study contributes to existing fields in the following parts. First, multiscale time heterogeneity PLRs between steel raw materials and steel products are considered. Second, by introducing the Podobnik test method, this study improves the cross-correlation result of calculating PLRs. Third, PLR structural features in the whole industry chain are discussed by utilizing a complex network analysis method, which detects more complicated steel market information.

The study is organized as follows: Section 2 is the literature review. Section 3 presents the data and method section. In this section, we introduce the data we used and method theory and equations. Section 4 presents the results and discussion. Here we present the calculation results and their meaning. Section 4 is the conclusion section, where we conclude this paper and extract the results.

## 2. Literature Review

In 2016, China imported more than 1.02 billion tons of iron ore and the country’s external dependence on iron ore reached more than 80% according to the China Iron and Steel Industry Association. Even though these days China’s booming economy begins to slow down, its GDP is almost 9.4 times that of 20 years ago, and the country’s total planned investment in real estate development enterprises in 2016 was 18.26 times that in the year 2002 (National Bureau Statistics of China, http://data.stats.gov.cn/easyquery.htm?cn=C01, accessed on 20 June 2022). Large steel consumption not only emits much CO_2_ into the atmosphere [20,21] but also causes dust pollution, especially air pollution, in the environment [22,23]. The steel industry is an integrated industry chain that originated from original iron ore and was processed into products like billets, wire rods, rebar, and others. In the tightly related steel industry chain, the demands of one particular product will severely influence others. This influence will be reflected in price variation. The price variation of materials, namely iron ore, will be severely transmitted to steel products.

For steel companies, raw materials prices significantly influence steel company profits [24]. Production technique and process, environmental and carbon emission pressure, and costs are critical factors when these companies decide which material to choose. Steel scrap has been used as material to produce steel products and can be added in the iron smelting phase to increase molten iron output [25,26]. Efficient steel scrap use will upgrade the sustainability of the steel industry and improve the economic benefits [27]. Compared to iron ore, scrap products accord more with the needs of sustainable development and low carbon production [28,29]. Compared with primary steel production, utilizing scrap steel as material produces up to three times less emissions [3]. However, iron ore is more cost-efficient for steel companies. Therefore, in the steel industry chain, steel companies face trade-offs in choosing materials between iron ore and steel scrap. Owing to China’s policies of cutting overcapacity in 2016 and banning substandard steel in 2017, much scrap steel emerged in the steel market, thus causing scrap steel prices to decrease and leading to a decline in costs for steel companies. Recently, facing stricter environmental pollution and the “carbon peak, carbon neutralization” target, steel companies have begun to find an environmentally friendly and cost-efficient way to address the situation [30]. Xuan and Yue [26] predicted that before 2020, scrap demand in China was lacking, and after 2020, the scrap ratio, namely the recycled level of scrap steel, would grow all the time.

As two important steel-making materials, scrap steel and iron ore both play important roles in influencing midstream and downstream steel prices and even the macroeconomy [25,31,32]. However, these studies do not go further to distinguish between the particular influence of iron ore and that of steel scrap. In this study, we take both iron ore and steel scrap into consideration as the material of steel products. Then we study how these materials influence steel product prices. Faghih and Kashani [33] used the vector correction model and Granger causality test method to test the relationship between materials. The result shows us that the price of iron ore explains and forecasts the price of steel.

Moreover, we want to study the time scale heterogeneity problem between the steel material-product PLRs. Ma and Wang [5] talk about the time-varying price dependencies, but they use steel company stock price as a proxy of the downstream steel industry, rather than the product prices which directly influence the steel industry chain. Therefore, we want to focus more on the industry chain products PLRs and their time scale heterogeneity. We utilize the MODWT method to decompose the original steel material and products price series into short-term, midterm, and long-term time scales to capture the multiscale price series. Jammazi [34] uses the wavelet cross-correlation method to calculate the lead-lag relationship between oil and stock index in six different countries among different time scales. Fang, Lu [35] use a multi-fractal detrended cross-correlation method to study the nonlinear fluctuation relationship between carbon emission allowance and stock returns in the European and Chinese markets. Compared to traditional econometric lead-lag relationship methods like Granger causality analysis, the combination of cross-correlation method and wavelet decomposition method can get richer information about lead-lag relation in different lag orders and time scales. Additionally, network analysis can be perfectly combined with price time series analysis to detect the overall characteristics and specific importance of steel material and product PLRs. Guo, Li [36] combine the Granger causality method and complex network method to calculate the price transmission activities among midstream steel products worldwide. The combination can be applied in other markets like the crude oil market [37,38], stock market [7], gold market [39], and macro-economy market [40].

## 3. Materials and Methods

Research data are collected from the Resset database. We use the Platts iron ore index and scrap steel price to represent the steel material price variance trend. We selected 16 steel products to melt and produced midstream and downstream steel products. The daily data range changes from 28 December 2012 to 29 September 2017. For missing data, this study averages the data between two dates.

Table 1 shows the 16 steel products in the steel industry chain. The first column shows the codes of the 16 steel products, and the second column shows the detailed steel product specifications. To obtain stable data, we obtain the return series $Y_{t}$ of each product.

(1) $$Y_{t}=log\left(s_{t}/s_{t-1}\right)$$

where $s_{t}$ is the daily price of each steel product price series. In addition to iron ore and steel scrap, the 16 steel products include billet, wire rod, plates of middle thickness, low-alloy plate, rebar, hot-rolled coil, cold-rolled coil, HSLA (High-strength low-alloy steel) plates, hot galvanized coil, Industrial & P round steel, and color coated board, besides iron ore and steel scrap. These 16 steel products include different stages in the steel-making process and cover different end-use demands in the economy. We take these 16 steel products into the dataset, aiming at studying the PLRs from raw materials to the whole steel industry chain.

### 3.1. Wavelet Transform

By introducing the maximal overlap discrete wavelet transform (MODWT) method, it is possible to decompose the original steel materials and products price data into different time scale series. These different time scales data reveal the implicit time-frequency domain information hidden in the original data. Compared to other wavelet methods, this wavelet transform method has the following advantages. First, the general discrete wavelet (DWT) method requests a data length equal to 2^n^ (n is a positive integer). The MODWT method does not make this demand. Second, through this method, we can attain more information from low-frequency series.

We divide the original data series into six different time-scale series. The equation is listed as follows [6,7]:

(2) $$X_{t}=\sum_{j=1}^{J_{0}}D_{J_{0}}+S_{J_{0}}$$

In this formula, $X_{t}$ represents the original time series data. j, which changes from 1 to $J_{0}$, and represents that the time scale of the series is $J_{0}$. Here, we set $J_{0}$ as 6. Tiwari, Oros [41] prove that six wavelet transform scales are suitable to gain adequate information from the time series data. $D_{J_{0}}$ represents the wavelet transform coefficient between scale $2^{j}\;\mathrm{and}\;2^{j+1}$ ($j=1,\ldots ,J_{0}$).

Table 2 shows the time scale range and their fluctuation range. Scale 1 level represents the time range from 2 to 4 days (daily effects), and Scale 2 level represents the time range from 4 to 8 days (weekly effects). These two scales are regarded as short-term level. Scale 3, whose range changes from 8 to 16 days is a medium-term scale. Additionally, Scale 4, which represents 16 to 32 days (monthly effects), is also a medium-term scale. Scale 5, whose time range changes from 32 to 64 days, and Scale 6, whose time range changes from 64 to 128 days, represent long-term levels.

### 3.2. Lead-Lag Relationship Calculation and Test

At different time scales, we use the cross-correlation method to calculate the lead-lag relationship between steel product prices and iron ore and steel scrap prices. The cross-correlation of two series is the correlation of these two time series at different time orders. In this research, the unit of time order is a day. The format is as follows [42]:

(3) ρXY(k)=cov(Xt,Yt+k){var(Xt)var(Yt)}12

where k is the lag order of cross-correlations. $X_{t}$ and $Y_{t+k}$ are time series of the research object. $\rho_{XY}$ is namely the influence degree that $X_{t}$ imposes on $Y_{t+k}$. In the research, we select the maximum or minimum account of a cross-correlation series between two steel product prices series as subjects. Lag order range is usually decided by theoretical considerations and industry knowledge [43].

When using the cross-correlation method, researchers could not find a proper method to test the results until Podobnik, Grosse [10] designed a testing approach based on Ljung-Box *Q* test method. The statistic of the Ljung-Box *Q* test [44] is:

(4) $$Q\left(m\right)=n\left(n+2\right)\sum_{k=1}^{m}\frac{\hat{\;\rho_{XY}\left(k\right)}\left(x_{k}^{2}\right)}{n-k}$$

where Q(m) obeys chi-square distribution with degree of freedom *n*. $\rho_{XY}\left(k\right)$ represents the autocorrelation coefficient of the random variable $X_{k}^{2}$ lagging k order and n represents the length of the random variable $x_{k}$. Based on statistic Q(m), Podobnik, Grosse [10] constructed a statistic $Q_{xy}\left(m\right)$.

(5) $$Q_{xy}\left(m\right)=n^{2}\sum_{k=1}^{m}\frac{\rho_{XY}^{2}\left(k\right)}{n-k}$$

where $ccr_{x\left(k\right)y\left(k\right)}$ represent the cross-correlation relationship between random variables $\left\{X_{t}\right\}$ and $\left\{Y_{t}\right\}$, k represents the corresponding order of cross-correlation relationships, m represents the degree of freedom, n represents the length of the random variables $\left\{X_{t}\right\}$ and $\left\{Y_{t}\right\}$. When n is large enough, $n^{2}\approx n\left(n+2\right)$. Equation (4) shows that $Q_{xy}\left(m\right)$ is similar to the chi distribution $\chi^{2}\left(m\right)$ with m degree of freedom. Podobnik, Grosse [10] calculate the statistics of cross-correlation relationships according to statistic $Q_{xy}\left(m\right)$ and compare them with the value of chi square distribution $\chi^{2}\left(m\right)$ at significance level of 0.05, if the values $Q_{xy}\left(m\right)$ > $\chi^{2}\left(m\right)$, then the cross-correlation relationship at order m is significant.

### 3.3. Complex Network Method

Based on calculation results, this study establishes the lead-lag relationship networks of steel products at different time scales by introducing the complex network method.

By introducing network indicators and analyzing the network topological features, structural features of the lead-lag relationship between steel materials and products at different time scales can be detected. In the network, nodes represent all commodities, including two steel materials and steel products. Edges represent price lead-lag relationships between different commodities, and width of edges represents the size of relationship coefficients. In the study, we introduce weighted out-degree and weighted out centrality closeness to study the price influence of two steel materials: iron ore and scrap steel.

#### 3.3.1. Weighted Degree

Weighted degree measures the sum of lead-lag relationship coefficients from one product to another. A larger weighted degree represents a larger price lead relation from one product to another, which indicates that the early price change of one product has higher relation with the later price change of other products. This indicator is represented as follows [45]:

(6) $$wk_{i}^{out}=\sum_{j=1}^{N}r^{ij}$$

where $wk_{i}^{out}$ represents the sum of price lead relations from product i to others, i and j represent different steel products, $r^{ij}$ represents the price lead-lag relationship between products i and j, N represents the number of nodes, namely products in the network.

#### 3.3.2. Out Closeness Centrality

Out closeness centrality measures the network distance from one product to others. This indicator represents how long it will take when the price lead relation transmits from one product to other reachable products through the shortest paths in the network [46].

Larger out closeness centrality means a closer distance between one product and others. Therefore, in the price lead-lag relationship network, price variation of this product more easily leads other products because of the close distance. The following equation shows how to calculate the average distance and closeness centrality [47].

(7) diout=1N∑j=1Ndij

(8) CCi=1diout=N∑j=1Ndij

Term $d_{i}$ represents the average distance between node i and all other nodes. Term $d_{ij}$ represents the shortest distance between node i and node *j*. Term $CC_{i}$ represents closeness centrality of the network, which is the reciprocal of $d_{i}^{out}$ [29]. A larger magnitude for the $CC_{i}$ means that node *i* is closer to other nodes in the network and node *i* is the center of the network.

This section may be divided by subheadings. It should provide a concise and precise description of the experimental results, their interpretation, and the experimental conclusions that can be drawn.

## 4. Results and Discussion

### 4.1. Data Description Analysis

**Figure 1:**
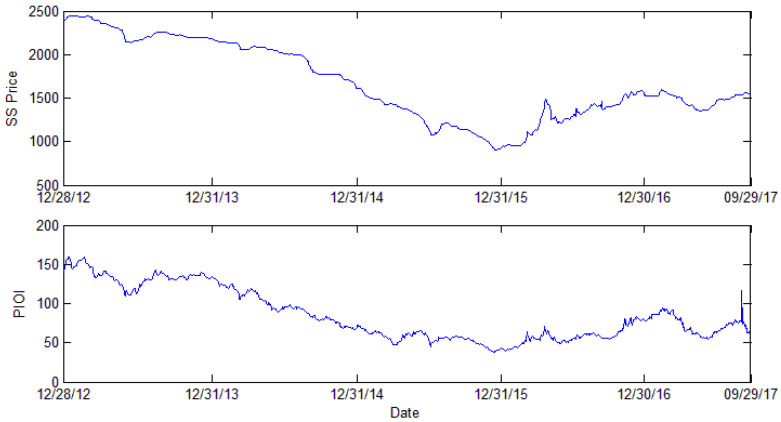
Steel scrap price and Platts iron ore index fluctuation.

Figure 1 shows the variation trend of steel scrap price and the Platts iron ore index. Figure 1 shows that the tendency is quite similar: From 12 December 2012 (2394.69) to 8 June 2013 (2138.75), the steel scrap price was always going down. After a two-month increase from 8 June 2013 (2138.75) to 22 August 2013 (2262.34), the steel scrap price fluctuated slowly until 17th December 2015 (905.63). In the former part of 2016, there was a sharp growth in steel scrap prices, which reached 1487.5 on 25 April 2015. Then, the price decreased and fluctuated at a relatively stable level. In 2017, the steel scrap price decreased to 1353.13 on 27 May 2017, and then rose to 1551.88 on 19 September 2017, the end of the sample period.

The steel scrap price is closely related to environmental policy and whole steel market performance. In the former part of 2013, the steel scrap price experienced a downturn until 8 June (2138.75). Then, the price climbed to 2262.34 on 22 August 2013. Then, the steel scrap price underwent a two-year depression until 17 December 2015, and the steel scrap price reached 905.63 yuan per ton. However, in the following 4 months, the steel scrap price increased to 1487.5 on 25 April 2016. Then, the price suddenly dropped to 1217.5 on 3 June 2016. Then, the steel scrap price rose along with fluctuations. In 2017, the scrap steel price experienced a decreasing and increasing period.

The Platts iron ore index uses the spot price, which is based on 62% Fe iron ore CFR (cost and freight) prices of the Qingdao steel market, a major China steel market. The spot price can better reflect the fluctuation tendency of the iron ore index and is widely used by steel market individuals, such as steel-making companies, traders, and mining companies. In June 2013, the PIOI index reached its lowest point (110.75) in 2013 and then climbed to 142.5 on 14 August. Subsequently, the PIOI index fluctuated to decrease until it reached the lowest point (38.5) in the sample period on 15 December 2015. In 2016, iron ore trading began to recover, and during the whole year, the PIOI index increased slowly except for the period from 21 April (70.5) to 9 May (54.85) and the period from 23 August (62.5) to 20 September (55.1). In 2017, the rising tendency continued until 21 February (95.05). Then, there is a sharp decline from 21 February (95.05) to 13 June (54). Then, the index increased, and on 7 September, there was a dramatic increase. The index was up to 116. Then, the PIOI fluctuated and fell.

In the former part of 2013, the steel material market fluctuated in a normal economic cycle. After the Chinese Spring Festival, the launched “Country of Five” policy reduced the expectation of the real estate market. Moreover, excessive inventory in iron and steel enterprises also worsened the situation. The upstream steel material market, therefore, declined. In July 2013, the steel material market began to recover, and expectations decreased a few months ago, thus causing the steel material supply to be tight. Macroeconomic statistical data issuance at this time also strengthened steel companies’ confidence in increasing production. From August 2013, however, the major international iron ore companies greatly improved their production capacity, while in China, this major iron ore consumer greatly reduced its steel production capacity because the environmental regulation in Hebei and Jiangsu Provinces curtails the steel capacity. During 2014, steel material demands in Asia, especially China, decreased. In 2015, a new environmental protection law was enforced. Ambiguous real estate prospects and the enactment of environmental protection policy reduced the capacity of steel enterprises and market expectations, which showed a lack confidence in 2014 and 2015, caused steel material to weaken in demand, and the market went into a downturn for these two years.

In 2016, as steel industry regulations such as cutting-capacity policies came into effect, steel enterprises started making profits again. Downstream demand upsurges led to steel product price increases, as did steel materials. However, in late April, a sudden steel price reduction led the amount of steel material market trading to shrink. Later, continuing market recovery and enterprise production activity caused a rebound in the market. In 2017, the downstream, construction-related demand driving force was limited. Other transportation infrastructure establishment stirs up the market expectation, but it takes time to test the actual economic effect. Upstream, China severely strikes substandard steel and medium frequency furnace steelmaking, considerable steel scrap flows into the market, and the steel scrap price greatly decreases. Like the price of steel scrap substitutes, the iron ore price also decreases. As backward capacity continues to be eliminated, the steel material market begins to run within a reasonable range. Concerning market supply and demand, the steel market is still recovering.

However, does the steel material price lead to the steel product price, or does the steel product price lead to the steel material price on earth?

**Figure 2:**
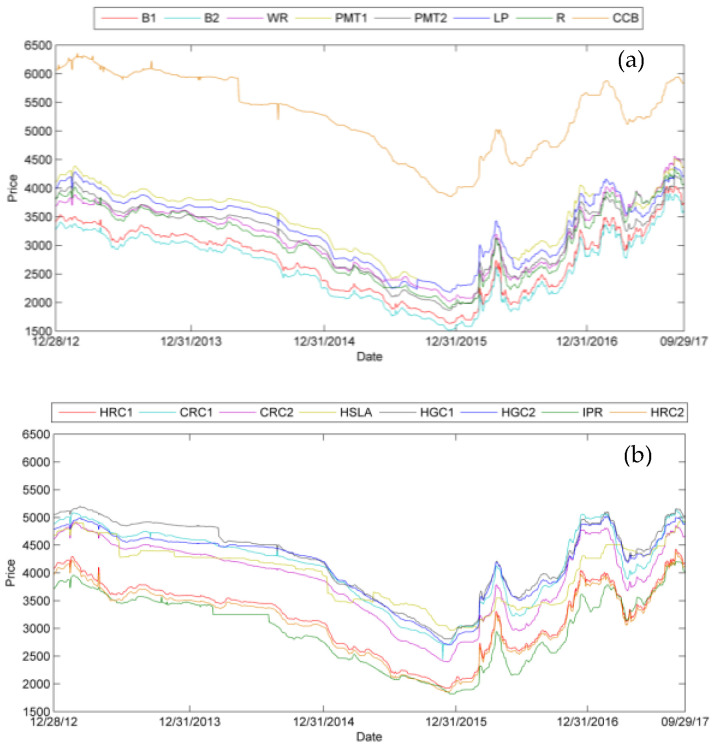
Midstream and downstream steel product price fluctuation.

In the actual steel market, as the most vital downstream phase, steel industry development influences the steel material price. The steel products connect the upstream material and downstream demand. Sixteen midstream and downstream steel product prices are presented in Figure 2.

Figure 2a,b show different steel product price fluctuation trends. Overall, the price variation represents a similar trend to steel material variation. Namely, from 2013, steel demand decreases. Environment regulation activity curtails industry production, and cutting-capacity activity eliminates backward steel capacity. The whole industry goes into a downturn. Until 2016, when the industry regulation in the past few years came into effect, the external economic environment also improved to a stable level, and the steel product price started to rebound. Prior to 2016, infrastructure construction was put into practice and reducing steel inventories pushed steel prices to grow. Steel product price growth gave the market individual confidence about market recovery, and this confidence accelerated the price surge again. However, these short-term soars lacked stable market fundamentals, and product prices tumbled considerably to a reasonable range. Then, the steel product price gradually increased as domestic and foreign demand recovered. In 2017, the real estate market was ambiguous, and it decreased the downstream demand. Additionally, this year, China struggled to eliminate substandard steel. Regulation improves the product quality and increases the enterprise profits. Therefore, there was a down and uptrend in 2017.

**Figure 3:**
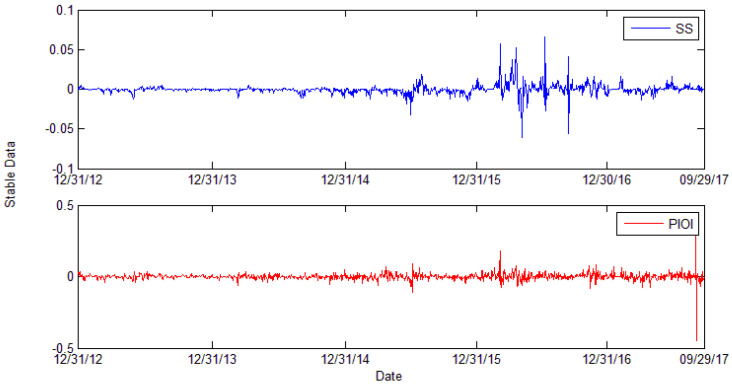
Stable series of scrap steel and Platts iron ore index series.

**Figure 4:**
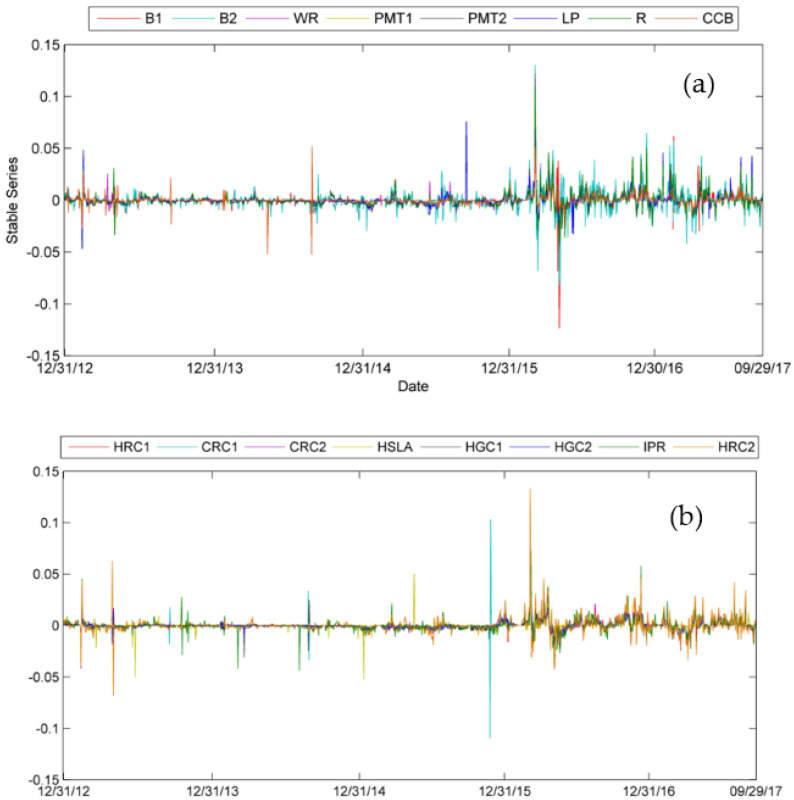
Stable series of 16 midstream and downstream steel products series.

We calculate the log return of the price series and attain the stable data series. The scrap steel price and Platts iron ore index log return series are presented in Figure 3. Midstream and downstream steel product prices log return series are presented in Figure 4.

In Figure 3, the blue line of SS represents the log return series of scrap steel. The red PIOI line represents the log return series of Platts iron ore index. The price of steel scrap fluctuates severely in the midterm of 2015 and the year 2016. The variation tendency of the Platts iron ore index is moderate. However, in the midterm of 2015 and early 2016, the index fluctuates a lot. In Figure 4a,b, different colors represent different midstream and downstream steel products.

### 4.2. Wavelet Decomposition Comparison

The wavelet decomposition method provides us with a perspective to capture the steel material and steel product price variation at different time scales. The detailed wavelet decomposition results are listed at Appendix A.

In the Appendix A, after decomposition of the original steel scrap price and Platts iron ore index, t in short-term, these two materials display various fluctuation patterns, while as the time scale grows, the fluctuation tendency gradually becomes concordant.

The steel product wavelet decomposition also exhibits similar features. This reflects that in the short-term, a particular steel product may fluctuate according to different reasons and represent various volatility tendencies. As time scale grows, those products are influenced by external factors as a whole steel market. Their fluctuation in the long run is in accordance with each.

However, there also exists distinguished features between steel products, especially in the short-term. In the scale 1 picture which depicts the price volatility within 2–4 days, these products fluctuate a lot at a particular date. This reflects that in the short-term, especially at 2–4 days period, these different steel products fluctuated according to their respective supply and demand rules.

Wavelet decomposition results reveal different time domain information for different market practitioners. For speculative investors who focus on short-term price fluctuations, it is critical to find the key price lead indicators because of the severely fluctuated markets in the short-term. For steelmaking companies, midterm and long-term price trend is the crucial time domain they should pay attention to. Their production and inventory decision are relatively long processes. Excessive attention on short-term results will disturb their normal operating activities. For policy makers, long-term time scale results should be paid more attention.

### 4.3. Price Lead-Lag Relationships Analysis

Then this study calculates the *p* values of the Podobnik test and results show that all the cross-correlation at six time scales passes the test. The detailed lead-lag relationship coefficients and their lag orders are shown in Table 3.

Table 3 shows the lag order results of PLRs between steel scrap and 16 steel products. The first column refers to the steel products code. The second column refers to the lag order results of original series. The last six columns refer to the lag order results of PLRs at six time scales. At original series, all lag orders of the PLRs are 0, which means that scrap steel price variation does not lead or lag the price fluctuation of steel products. Their price variation occurs in the same day. But at scale 1, the lag order results fluctuate a lot. PLR lag orders do not present unified rules that can be detected. This may be because at short-term, investors’ decisions are influenced by short-term market information and change quickly. Market volatility is high. At scale 2, most PLR lag orders become 0, which means scrap steel price and steel product prices fluctuate on the same day, but PLRs “Scrap steel to HRC1” and “Scrap steel to CRC1” steel present that scrap steel price lags HRC1 and CRC1 for 116 and 344 days respectively. This may because of the mediation of hot rolled coil and cold rolled coil products in the steel industry chain. These two steel product prices will in turn lead upstream materials. At midterm of scale 3 and scale 4, scrap steel price lags products Billet/Color coated board/Industrial round steel for one lag order. At long term, scrap steel price still lags billet products, but PLRs between scrap steel and other products differ according to steel products. In scale 5 (32–64 days), scrap steel price leads most steel products except Billet products/Low-alloy plate/Rebar/Color coated board/Industrial & P round steel. The leading lag order ranges from −4 to −1, which means at long term, scrap steel price leads these products for 32–256 days. What’s more, Low-alloy plate/Rebar/Color coated board/Industrial round steel prices fluctuates at the same lag order with scrap steel. At scale 6, in all 16 steel products, those products which are located the in upper stage of the industry chain, such as Billet/Wire rod/Plates of middle thickness/Rebar product prices lead the scrap steel price. Those which are located at a further downstream stage of the steel industry chain, such as Hot rolled coil/Cold rolled coil/High-strength low-alloy plate/Hot galvanized coil product prices lag scrap steel. These products endure a relatively long smelting process and their final application is more narrow than that of upper stage products. Therefore, their long-term price influence is weak.

The lead-lag relationship between Platts iron ore index and steel products also tells a similar story. Table 4 shows the lag order results of PLRs between Platts iron ore index and 16 steel products. The first column refers to the steel products code. The second column refers to the lag order results of original series. The last six columns refer to the lag order results of PLRs at six time scales. The original series PLR lag order results are almost at −1, except the Color coated board product (at lag 785) and Cold rolled coil 0.5mm product (at lag 460). The results denote that Platts iron ore index usually fluctuates with the steel products at the same time. However, for Color coated board products and Cold rolled coil 0.5mm products, the Platts iron ore index usually lags them for more than one year. Moreover, Platts iron ore index and hot rolled coil products fluctuate at the same lag order. At scale 1, iron ore price variation lags 16 steel products, and the lag order results are extremely large. The reason may be the same as scrap steel’s scale 1 lag order results. In short-term, the investors’ decisions are influenced by short-term market information and change quickly. Market volatility is high. This trend continues to scale 2. In scale 2, Hot rolled coil 3 mm/Cold rolled coil 0.5 mm/High-strength low-alloy plate/Hot galvanized coil 1 mm products are still extremely large, but other products’ lag order results become normal. Iron ore price leads these steel products for one lag. At midterm scale 3 and scale 4, iron ore price leads almost all steel products except Cold rolled coil 0.5 mm product. On the one hand, results show the strong price lead influence of iron ore. On the other hand, Cold rolled coil product can in turn influence iron ore because of its importance in the industry chain. At long term scale 5, iron ore price leads all 16 steel products, and at scale 6, Billet products/Color coated board/Industrial & P round steel prices fluctuate at the same lag order with iron ore price, while other product prices fluctuations still lag the iron ore price. At long term, iron ore’s price no more lags Cold rolled coil products. Different from other products, PLR lag orders between iron ore index and Cold rolled coil product prices still fluctuate at long term and present extremely large results.

The lead-lag relationship results show that steel scrap price is tightly related to steel products, and their lead-lag relationship usually transmits with a lag order, namely one day. For Platts iron ore index, things are a bit different. Most steel products are related to the Platts index, while Color coated board products and Cold rolled coil 0.5 mm product prices lead the index by 785 and 460 days respectively. For other products, except that they have the largest lead-lag relation coefficients with Platts index at −1 lag order, they also present relatively strong lead relationships at other lag orders.

Comparing steel scrap to the Platts iron ore index, we can find that steel scrap price usually fluctuates with steel products at the same time. What is more, all the lead-lag relation is positive. However, for Platts iron ore index, its variation advances most steel products. This may be because iron ore, as the major material in the steel market, influences the steel scrap market and steel products market a lot. For steelmaking companies, they should focus more on iron ore price variations in the market because iron ore price variation leads most steel products for one day. This can help related companies regulate their production decision and hedge the price fluctuation risk in advance. However, Hot rolled coil product price variation lags the upstream iron ore price variation. This may because of hot rolled coil product’s vital mediation in the industry chain and it influences the upstream iron ore price in turn. This is a key price lead indicator for steel industries, because hot rolled coil product price leads the main steel materials.

What is more, the products (Color coated board, Cold rolled coil 0.5 mm, High-strength low-alloy plate) whose prices lead steel materials exist at the end of the steel industry chain.

Then we discuss the network results to find the structural price relationship in the steel market.

### 4.4. Complex Network Analysis

Then we establish steel products price lead-lag relationship network at each time scale and compare their network topological indicators. The industry chain compares two main steel materials.

**Figure 5:**
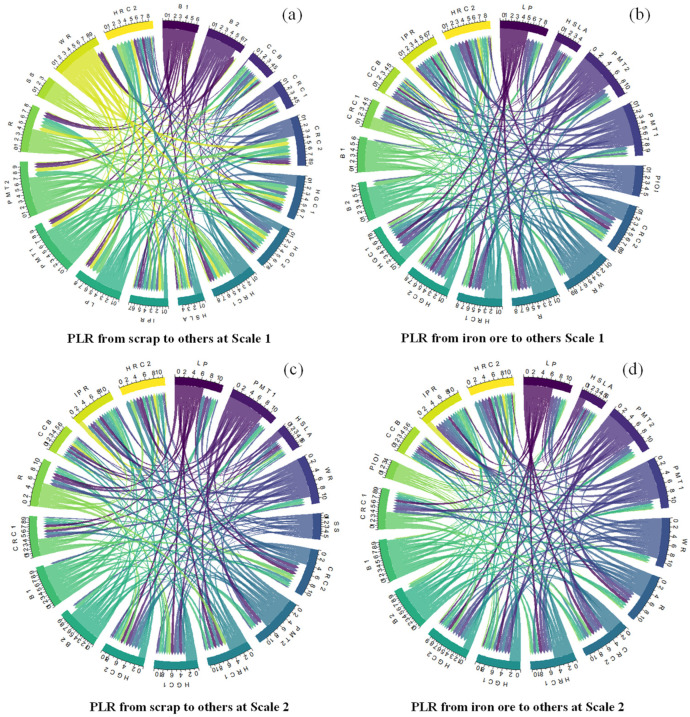

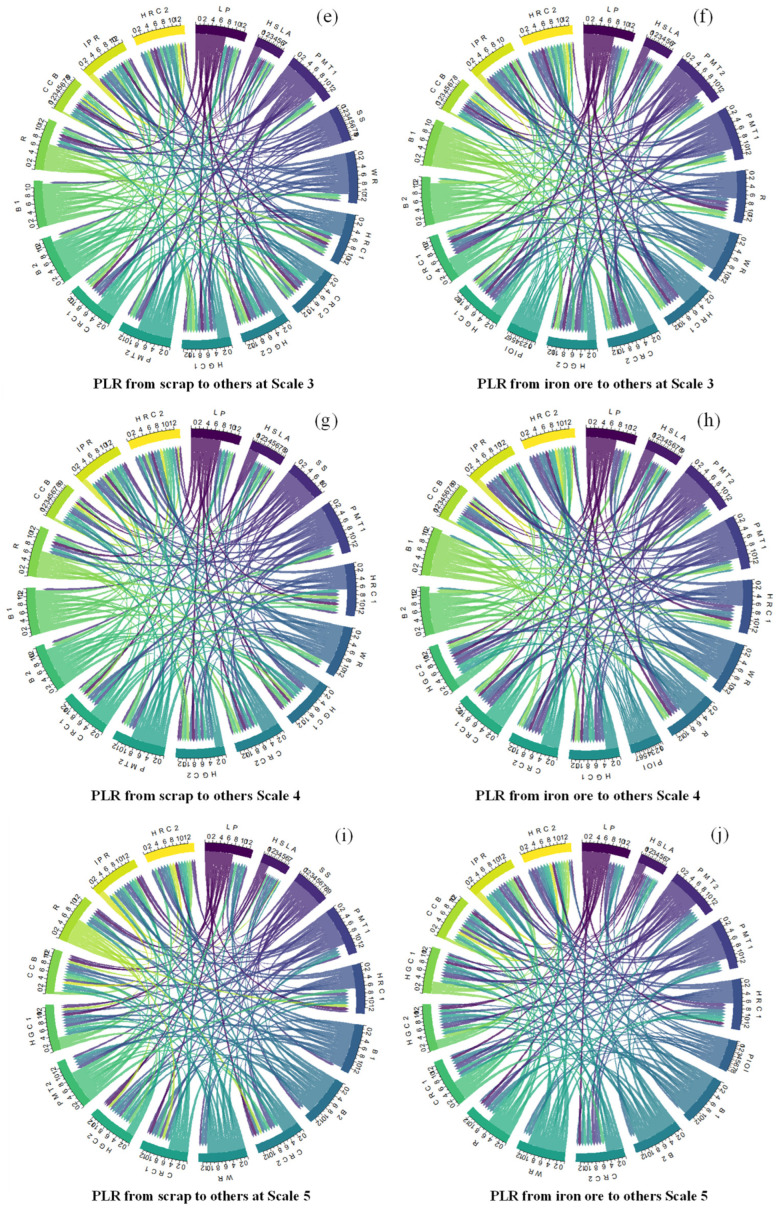

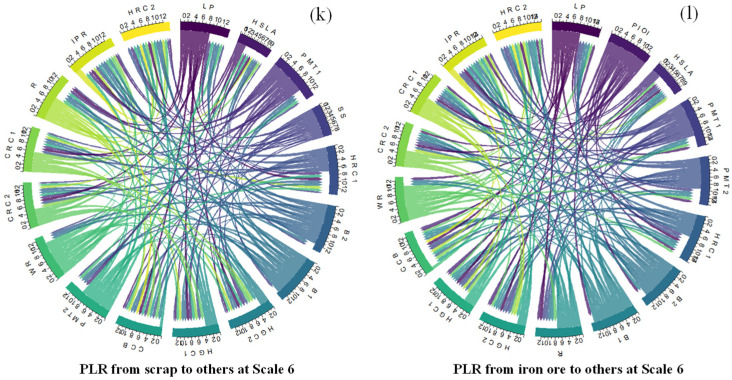
Price lead-lag relationship from iron ore and scrap steel to 16 steel products in 6 scales.

Figure 5 shows the PLRs from scrap steel and iron ore to other steel products at six time scales. Each color represents every steel product, and the flows with arrows between each entity represent the lead-lag relationship between steel products. The width of flow represents the number of lead-lag relationship coefficients between two steel products. Each arc is combined with two parts: those that lead relationships from one steel product to other products and those that lag relationships from other steel products to this entity.

Figure 5 reveals two trends in the PLRs from materials to steel products at different time scales. The first trend shows that as the time scale becomes longer, the PLR will be larger, which means that in long-term, there exists closer lead relationships from steel materials to steel products. In short-term scale 1, PLR from scrap to 16 steel products is −1.162, which means that price variation of scrap steel leads the downstream steel products and the summation of price lead relationship of all products is −1.162. At another short time scale 2, PLRs from scrap steel to downstream steel products have a large growth. The relationship coefficient is 5.946, while in long-term scale, the lead relationship is 8.903 (Scale 5) and 8.925 (Scale 6). The price lead relationship from iron ore to steel products also presents this trend. The price lead relationship in short-term is −3.895 (Scale 1) and 0.240 (Scale 2), while in long-term, the lead relationship is 8.532 (Scale 5) and 12.967 (Scale 6). The difference between short-term and long-term relationships may result from the relatively severe price fluctuation in short period. The market individuals cannot react to market fluctuations and enact corresponding measures in a short period. This causes a deviation between price signals and market fundamentals, so there presents the negative correlation between steel materials and products. As time scale grows and market individuals react to upstream price variation, the change in product price in the whole industrial chain shows a positive correlation trend. Similar results with the wavelet transform analysis are presented which prove the closer lead relationship from steel materials to steel products in long-term. For steelmaking companies, midterm and long-term price trend is the crucial time domain they should pay attention to. Their production and inventory decision are relatively long processes. Excessive attention on short-term results will disturb their normal operating activities.

The second trend shows that iron ore has tighter PLRs than downstream steel products at the shortest time scale and longest time scale, while scrap steel has tighter PLRs with downstream steel products at a relative midterm period. Iron ore price has a tight PLR with the downstream steel products in short-term of 2–4 days (−3.895) and long-term of 64–128 days (12.967). Scrap steel prices influence the downstream steel price at midterm of 8–32 days (7.557 and 6.599). At scale 2 (0.240) for short-term and scale 5 (8.532) for long-term, scrap steel also has tighter price relationships with steel products than iron ore. Table 5 shows the network indicator calculation results of each network.

Table 5 shows the weighted outdegree and out closeness centrality indicator of 12 networks. Each row shows the two network indicators at the scrap steel PLRs network and iron ore PLRs network at each time scale. The network indicator results show the same trends from the network analysis in Figure 5.

According to the out-closeness centrality indicator results, on the one hand, as the time scale grows, centrality of two materials declines gradually. In short-term, midterm and long-term scales, the average lead centrality from scrap steel materials to steel products is 0.211 (Scale 1/Scale 2), 0.106 (Scale 3/Scale 4), and 0.090 (Scale 5/Scale 6), while the indicators of iron ore are 0.207 (Scale 1/Scale 2), 0.129 (Scale 3/Scale 4) and 0.090 (Scale 5/Scale 6). This reflects that as the time scale grows, the network distance between materials and end-use steel products becomes short, which means the price variation of two materials is easier to impose on each downstream product. In short-term, price variation of steel materials is reflected in the iron ore and scrap steel spot trading market. Because of the time lag effects, steel products at midstream and downstream react to the market information slowly. As time scale grows, in the midterm and long-term, market individuals at midstream and downstream begin to regulate their supply and demand based on price information. This implication is consistent with the market activities. Then the price fluctuation in upstream materials transmits to downstream products. Therefore, in long-term, two steel materials are easier to lead end-use steel products. For steel companies, it is important to focus on this price transmission process, especially this vertical transmission from different segments of the industry chain. Little attention is usually paid to the horizontal regional price transmission activities. In long-term, upstream steel material prices are easier to lead downstream steel products. This indicates that for policy makers, policy regulation targets should be unambiguous, but policy regulation processes should be flexible. Policy regulation enacted by policy makers is aimed at long-term industry development and focuses on long-term price information. Therefore, the policy target should be unambiguous. In long-term, upstream steel material prices are easier to lead downstream steel products, but the short-term and midterm price trend may fluctuate and influence the speculative investors and steel companies’ decisions. When making long-term policies, public sectors should take market individuals’ response into consideration. Its policy regulation process should be flexible.

### 4.5. Robustness Test Analysis

After finishing the calculation and analysis, this study replaces cross-correlation method with another time series analysis method, called Granger causality test method, to make a robustness test in order to test the different time series analysis calculation results difference and their influence on final conclusions.

First the stationary test is enacted. This study uses Augmented Dickey-Fuller (ADF) and Phillips and Perron (PP) stationary test method. Results show all series at six time scales pass the stationary test at 0.05 significance level except some steel product prices in scale 6, which do not pass the PP test at 0.05 significance level but pass at 0.10 level. The PP test results of these series are showed in Table 6.

In Table 6, iron ore/Billet/Hot galvanized coil/High-strength low-alloy plate products at time scale 6 do not pass the PP test at 0.05 significance level but pass at 0.10 level. Then this study constructs a VAR model and calculates the Granger causality test results. Then, this study also constructs a Granger causality network of Scrap steel and Platts iron ore index at six scales. The network indicator calculation results are presented at Table 7.

Table 7 show the Granger causality analysis network indicator results. Because there are no weights in these networks, the degree results cannot reflect abundant information compared to the cross-correlation method. As time scale grows, degrees between scrap steel and steel products present the tendency of going up firstly and going down secondly. While the relationship between iron ore and steel products are relatively stable, the out closeness centrality results of iron ore relationship networks are also more stable than scrap steel relationship networks.

## 5. Conclusions

In the study, we focus on the multiscale price lead-lag relationships between steel materials and products. After decomposing the original data series into short-term, midterm, and long-term time scales series, we calculate the price lead-lag relationships through cross-correlation and filter the results by the Podobink test. At last, based on calculation results, this study constructed a price lead-lag relationship network to distinguish the structural features between two steel materials. The price relationship, which transmits across the industry chain, is usually paid little attention by industry practitioners, especially when there are two main upstream materials in the market. We attain the following conclusions.

First, most scrap steel and steel product prices fluctuate at the same time lag order. However, iron ore volatility leads following products for one day, such as Billet 20 Mnsi product, Billet Q235 product, Wire rod 6.5 mm product, Plates of middle thickness 8 mm product, Plates of middle thickness 20 mm product, Low-alloy plate 20 mm product, Rebar400, 20 mm product, Cold rolled coil 1 mm product, Hot galvanized coil 0.5 mm product, Hot galvanized coil 1mm product, and Industrial & P round steel Q235 product. Scrap steel lags Color coated board 0.476 mm product, Cold rolled coil 0.5 mm product, and High-strength low-alloy plate Q460 20 mm product for more than one year. It fluctuates with Hot rolled coil 3 mm and 4.75 mm product at the same time. Moreover, hot rolled coil product price is the key price lead indicator which should be focused on by all still industry practitioners.

Second, when steel price fluctuates, those downstream products usually lead to iron ore price. In the steel market, Color coated board 0.476 mm product, Cold rolled coil 0.5 mm product, and High-strength low-alloy plate Q460 20 mm product are at the last phase of industry chain, and individuals in the market should focus on these products. The price variation of these products will influence steel material prices.

Third, network indicator analysis shows that as the time scale becomes longer, there exist closer lead relationships from steel materials to steel products. Moreover, iron ore has tighter PLRs with downstream steel products in shortest time scale and longest time scale. In the long-term, two steel materials more easily lead end-use steel products.

## Figures and Tables

**Table 1:** 16 midstream and downstream steel products data explanation.

Code	Midstream and Downstream Steel Products
B1	Billet 20 Mnsi
B2	Billet Q235
WR	Wire rod 6.5 mm
PMT1	Plates of middle thickness 8 mm
PMT2	Plates of middle thickness 20 mm
LP	Low-alloy plate 20 mm
R	Rebar400, 20 mm
HRC1	Hot rolled coil 3 mm
CRC1	Cold-rolled coil 0.5 mm
CRC2	Cold-rolled coil 1 mm
HSLA	High-strength low-alloy plate Q460 20 mm
HGC1	Hot galvanized coil 0.5 mm
HGC2	Hot galvanized coil 1 mm
IPR	Industrial & P round steel Q235
CCB	Color coated board 0.476 mm
HRC2	Hot-rolled coil 4.75 mm

**Table 2:** Names of different time scales.

Time Scale	Fluctuation Range (Days)	Scale Name
Scale 1	2–4	Short-Term
Scale 2	4–8	
Scale 3	8–16	Medium-Term
Scale 4	16–32	
Scale 5	32–64	Long-Term
Scale 6	64–128	

**Table 3:** PLR lag orders between steel scrap and steel products at 6 time scales.

Steel Code	Original Series	Scale 1	Scale 2	Scale 3	Scale 4	Scale 5	Scale 6
B1	0	48	0	1	1	1	2
B2	0	−110	0	1	1	1	2
WR	0	0	0	0	0	−1	3
PMT1	0	532	0	0	0	−1	2
PMT2	0	89	0	0	0	−1	2
LP	0	136	0	0	0	0	0
R	0	89	0	0	0	0	4
CCB	0	136	0	1	1	0	0
HRC1	0	207	116	0	0	−4	−3
HRC2	0	89	0	0	0	−3	−3
CRC1	0	344	342	0	0	−4	−9
CRC2	0	89	0	0	0	−3	−1
HSLA	0	89	0	0	0	−3	−2
HGC1	0	137	0	0	0	−2	0
HGC2	0	532	0	0	0	−4	−2
IPR	0	89	0	1	1	0	0

**Table 4:** Lead-lag relationship between Platts iron ore index and steel products.

Steel Code	Original Series	Scale 1	Scale 2	Scale 3	Scale 4	Scale 5	Scale 6
B1	−1	349	−1	−1	−2	−2	0
B2	−1	389	−1	−1	−1	−2	0
WR	−1	190	−1	−1	−2	−4	−2
PMT1	−1	785	−1	−1	−1	−2	−2
PMT2	−1	389	−1	−1	−1	−2	−2
LP	−1	389	−1	−1	−2	−3	−2
R	−1	389	−1	−1	−2	−3	−1
CCB	785	389	−1	−1	−1	−3	0
HRC1	0	460	460	−1	−2	−6	−3
HRC2	0	389	−1	−1	−2	−5	−3
CRC1	460	597	1097	4	9	−52	−393
CRC2	−1	389	−1	−1	−2	−232	−5
HSLA	596	389	389	−1	−2	−8	−4
HGC1	−1	389	−1	−2	−3	−5	−3
HGC2	−1	785	785	−2	−3	−6	−5
IPR	−1	389	−1	−1	−1	−2	0

**Table 5:** Network indicators of price lead-lag network at different time scales.

Time Scale	Material	Weighted Degree	Out Closeness Centrality	Mateiral	Weighted Degree	Out Closeness Centrality
Scale 1	SS	−1.162	0.254	IORE	−3.895	0.176
Scale 2	SS	5.946	0.168	IORE	0.240	0.237
Scale 3	SS	9.002	0.111	IORE	7.557	0.132
Scale 4	SS	8.191	0.100	IORE	6.599	0.125
Scale 5	SS	8.903	0.102	IORE	8.532	0.117
Scale 6	SS	8.925	0.077	IORE	12.967	0.063

**Table 6:** PP stationary test results.

Steel Product Name	*p*-Value of PP Test
PIOI	0.064912
B1	0.050989
B2	0.051895
HSLA	0.070719
HGC1	0.061526
HGC2	0.066065

**Table 7:** Network indicators of Granger causality network at different time scales.

Time Scale	Material	Degree	Out Closeness Centrality	Material	Degree	Out Closeness Centrality
Scale 1	SS	4	0.023	IORE	14	0.056
Scale 2	SS	16	0.063	IORE	15	0.059
Scale 3	SS	6	0.036	IORE	13	0.053
Scale 4	SS	6	0.036	IORE	14	0.056
Scale 5	SS	12	0.050	IORE	16	0.063
Scale 6	SS	7	0.040	IORE	12	0.050

**Figure A1:**
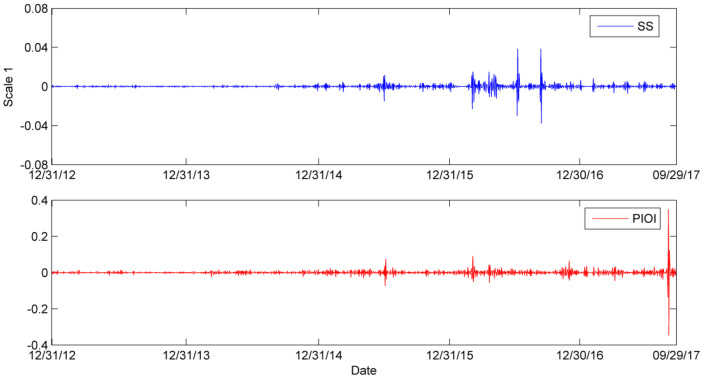
Wavelet decomposition results of steel materials prices series in Scale 1.

**Figure A2:**
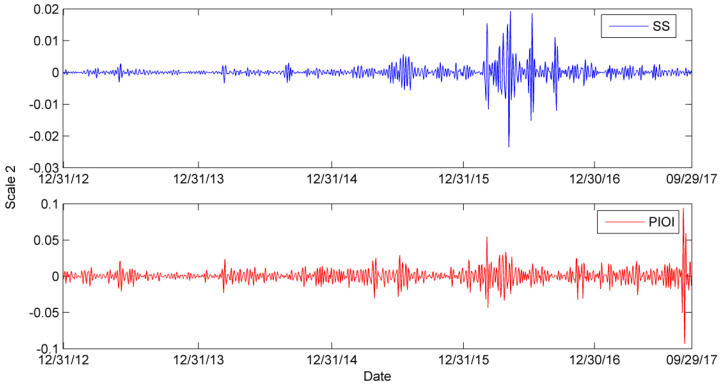
Wavelet decomposition results of steel materials prices series in Scale 2.

**Figure A3:**
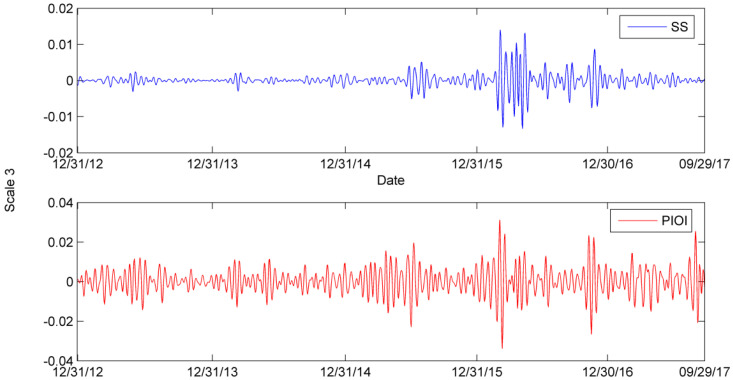
Wavelet decomposition results of steel materials prices series in Scale 3.

**Figure A4:**
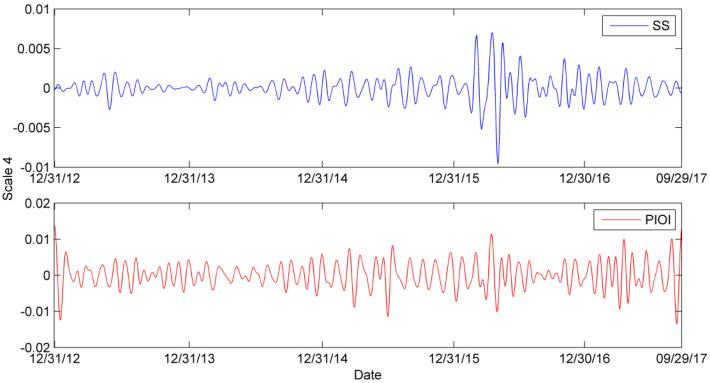
Wavelet decomposition results of steel materials prices series in Scale 4.

**Figure A5:**
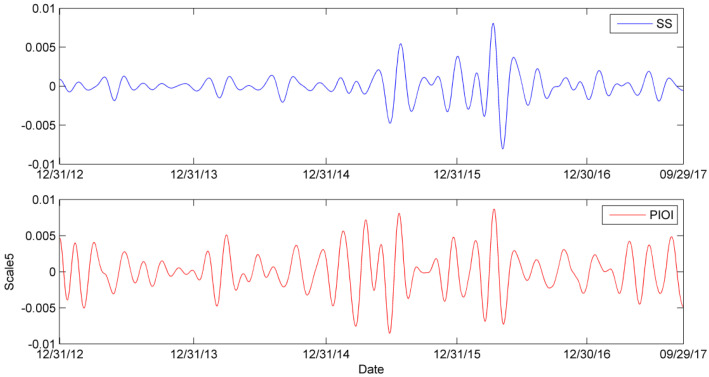
Wavelet decomposition results of steel materials prices series in Scale 5.

**Figure A6:**
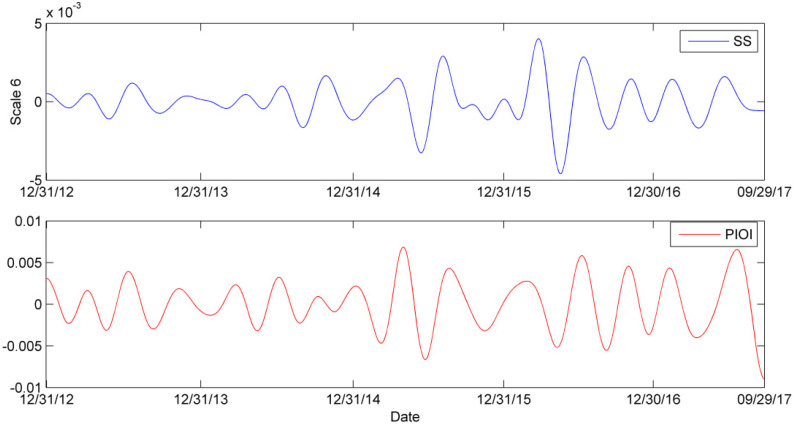
Wavelet decomposition results of steel materials prices series in Scale 6.

**Figure A7:**
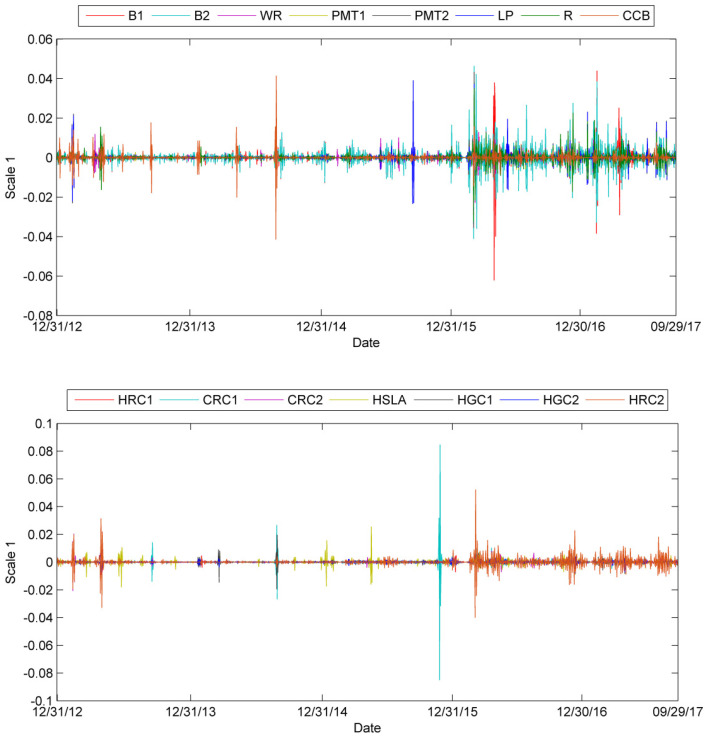
Wavelet decomposition results of steel products prices series in Scale 1.

**Figure A8:**
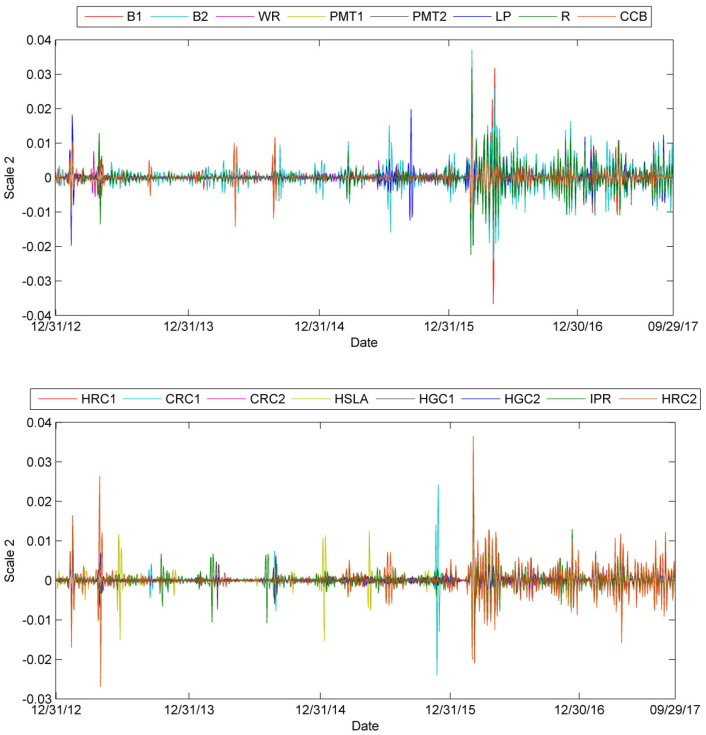
Wavelet decomposition results of steel products prices series in Scale 2.

**Figure A9:**
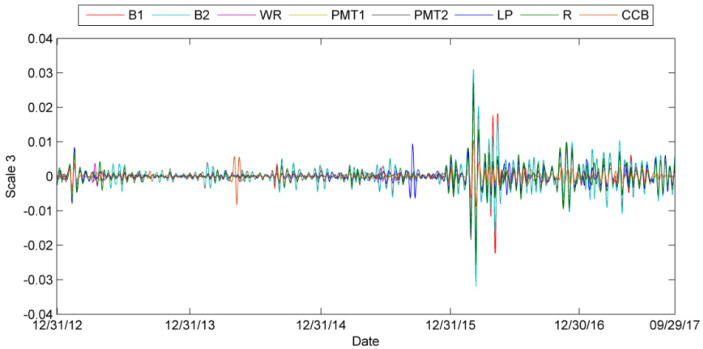

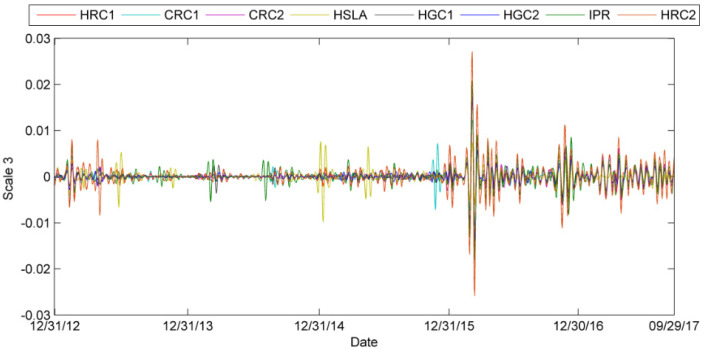
Wavelet decomposition results of steel products prices series in Scale 3.

**Figure A10:**
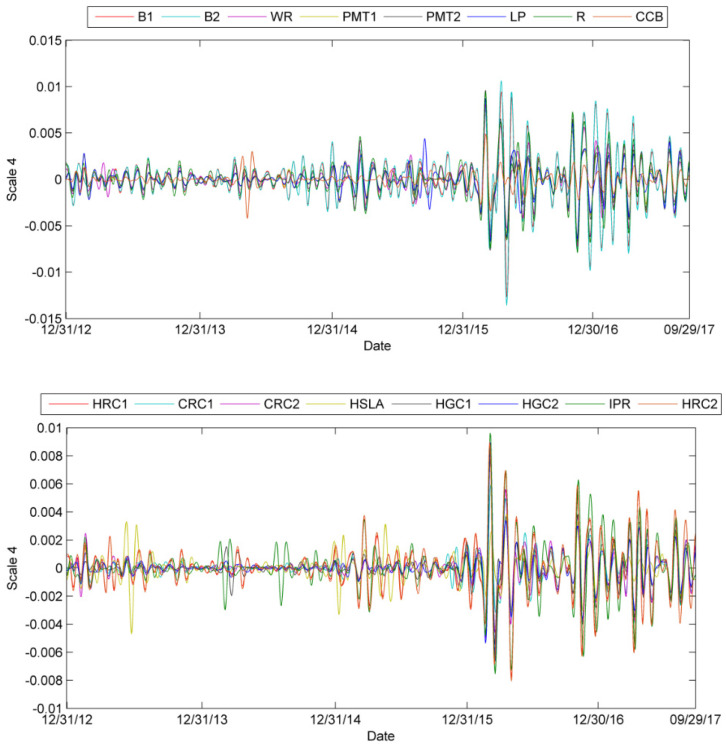
Wavelet decomposition results of steel products prices series in Scale 4.

**Figure A11:**
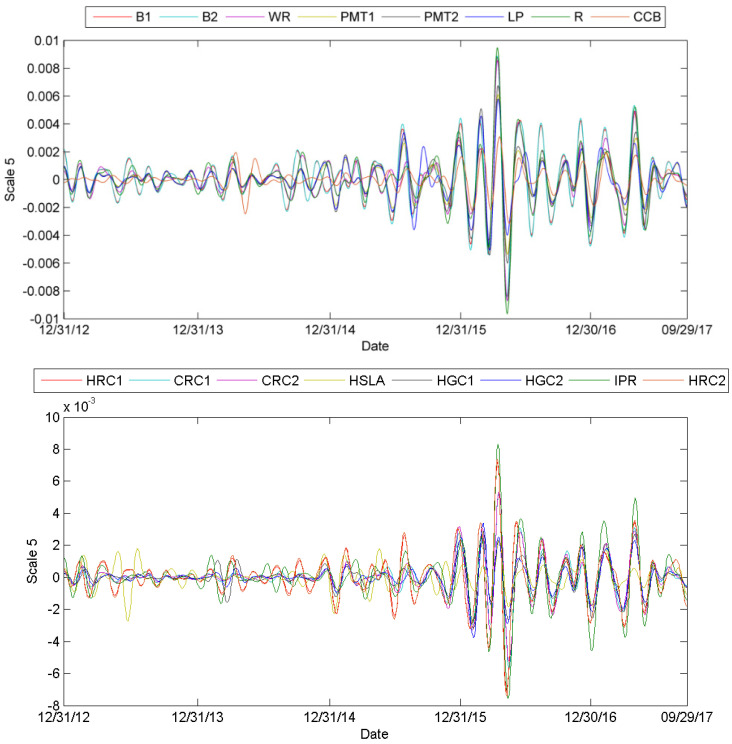
Wavelet decomposition results of steel products prices series in Scale 5.

**Figure A12:**
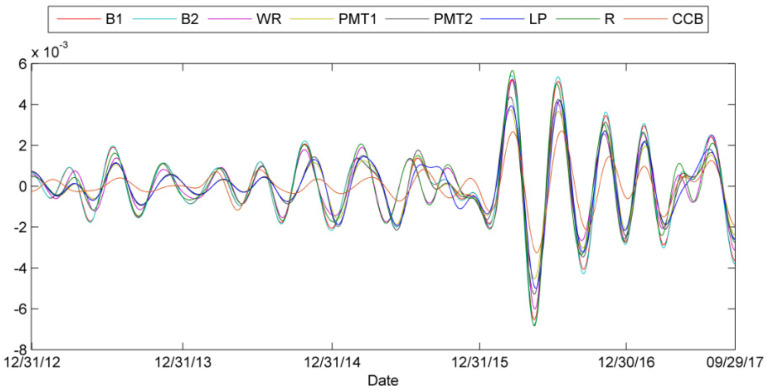

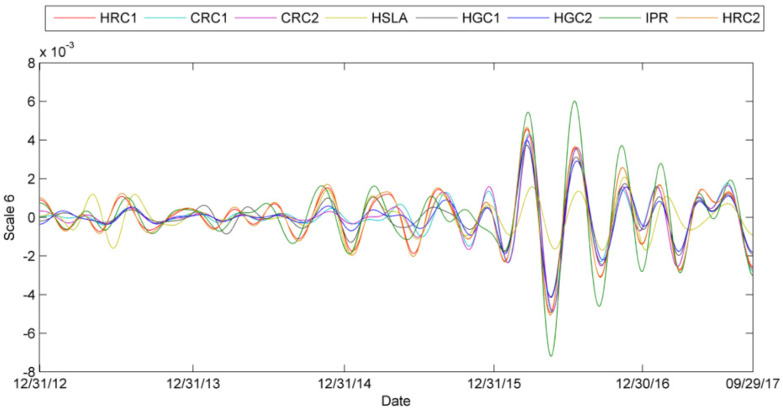
Wavelet decomposition results of steel products prices series in Scale 6.

## Data Availability

Data sharing not applicable.

## Appendix A

In this part, we list the wavelet decomposition figures from scale 1 to scale trend about 16 midstream and downstream steel materials and steel products. In the figures, SS represents steel scrap and PIOI represents Platts iron ore index. The code of different steel products explanation is listed as follows.

Figure A1, Figure A2, Figure A3, Figure A4, Figure A5 and Figure A6 present the Maximal overlap discrete wavelet transform (MODWT) decomposition results of scrap steel price and Platts iron ore index series. Figure A7, Figure A8, Figure A9, Figure A10, Figure A11 and Figure A12 present the maximal overlap discrete wavelet transform (MODWT) decomposition results of 16 midstream and downstream steel products prices series. There exists six time scale results. Scale 1 and Scale 2 represent short-term scale, Scale 3 and Scale 4 represent midterm scale, and Scale 5 and Scale 6 represent long-term scale. The blue line of SS represents the scrap steel wavelet decomposition series and the red line of PIOI represents the Platts iron ore index wavelet decomposition series.

## References

[1] Liu J., An R., Xiao R., Yang Y., Wang G., Wang Q. (2017). Implications from substance flow analysis, supply chain and supplier’ risk evaluation in iron and steel industry in Mainland China. Resour. Policy.

[2] Qi Y., Li H., Liu Y., Feng S., Li Y., Guo S. (2020). Granger causality transmission mechanism of steel product prices under multiple scales—The industrial chain perspective. Resour. Policy.

[3] Flint I.P., Cabrera Serrenho A., Lupton R.C., Allwood J.M. (2020). Material Flow Analysis with Multiple Material Characteristics to Assess the Potential for Flat Steel Prompt Scrap Prevention and Diversion without Remelting. Environ. Sci. Technol..

[4] Ma Y. (2021). Do iron ore, scrap steel, carbon emission allowance, and seaborne transportation prices drive steel price fluctuations?. Resour. Policy.

[5] Ma Y., Wang J. (2021). Time-varying spillovers and dependencies between iron ore, scrap steel, carbon emission, seaborne transportation, and China’s steel stock prices. Resour. Policy.

[6] Huang X., An H.Z., Gao X.Y., Hao X.Q., Liu P.P. (2015). Multiresolution transmission of the correlation modes between bivariate time series based on complex network theory. Phys. A Stat. Mech. Its Appl..

[7] Sui G., Li H., Feng S., Liu X., Jiang M. (2018). Correlations of stock price fluctuations under multi-scale and multi-threshold scenarios. Phys. A Stat. Mech. Its Appl..

[8] Polanco-Martinez J.M., Abadie L.M. (2016). Analyzing Crude Oil Spot Price Dynamics versus Long Term Future Prices: A Wavelet Analysis Approach. Energies.

[9] Miśkiewicz J. (2021). Network Analysis of Cross-Correlations on Forex Market during Crises. Globalisation on Forex Market. Entropy.

[10] Podobnik B., Grosse I., Horvatić D., Ilic S., Ivanov P.C., Stanley H.E. (2009). Quantifying cross-correlations using local and global detrending approaches. Eur. Phys. J. B.

[11] Watts D.J., Strogatz S.H. (1998). Collective dynamics of ‘small-world’ networks. Nature.

[12] Barabasi A.L., Albert R. (1999). Emergence of scaling in random networks. Science.

[13] Qazi R., Parker K.E., Kim C.Y., Rill R., Norris M.R., Chung J., Bilbily J., Kim J.R., Walicki M.C., Gereau G.B. (2021). Scalable and modular wireless-network infrastructure for large-scale behavioural neuroscience. Nat. Biomed. Eng..

[14] Avila A.M., Mezić I. (2020). Data-driven analysis and forecasting of highway traffic dynamics. Nat. Commun..

[15] Zhou W., Chen J., Ding B.Q. (2018). Optimal Flow Distribution of Military Supply Transportation Based on Network Analysis and Entropy Measurement. Entropy.

[16] Barucca P., Bardoscia M., Caccioli F., D’Errico M., Visentin G., Caldarelli G., Battiston S. (2020). Network valuation in financial systems. Math. Financ..

[17] Ding H., Jin Y., Liu Z., Xie W. (2019). The relationship between international trade and capital flow: A network perspective. J. Int. Money Financ..

[18] Zhang Y.T., Zhou W.X. (2021). Microstructural Characteristics of the Weighted and Directed International Crop Trade Networks. Entropy.

[19] Alves L.G.A., Mangioni G., Rodrigues F.A., Panzarasa P., Moreno Y. (2018). Unfolding the Complexity of the Global Value Chain: Strength and Entropy in the Single-Layer, Multiplex, and Multi-Layer International Trade Networks. Entropy.

[20] Liu S., Tian X., Cai W., Chen W., Wang Y. (2018). How the transitions in iron and steel and construction material industries impact China’s CO_2_ emissions: Comprehensive analysis from an inter-sector linked perspective. Appl. Energy.

[21] Lin B., Liu H. (2015). CO_2_ mitigation potential in China’s building construction industry: Acomparison of energy performance. Build. Environ..

[22] Ou J., Meng J., Zheng J., Mi Z., Bian Y., Yu X., Liu J., Guan D. (2017). Demand-driven air pollutant emissions for a fast-developing region in China. Appl. Energy.

[23] Wang Y., Lai N., Mao G., Zuo J., Crittenden J., Jin Y., Moreno-Cruz J. (2017). Air pollutant emissions from economic sectors in China: A linkage analysis. Ecol. Indic..

[24] Ou T.Y., Cheng C.Y., Chen P.J., Perng C.Y. (2016). Dynamic cost forecasting model based on extreme learning machine—A case study in steel plant. Comput. Ind. Eng..

[25] Omura A., Todorova N., Li B., Chung R. (2016). Steel scrap and equity market in Japan. Resour. Policy.

[26] Xuan Y., Yue Q. (2016). Forecast of steel demand and the availability of depreciated steel scrap in China. Resour. Conserv. Recycl..

[27] Huang Y.H., Huang Z. (2014). On Building a Push-Pull Supply Chain of Iron and Steel Scrap. Adv. Mater. Res..

[28] Ren M., Lu P.T., Liu X.R., Hossain M.S., Fang Y.R., Hanaoka T., O’Gallachoir B., Glynn J., Dai H.C. (2021). Decarbonizing China’s iron and steel industry from the supply and demand sides for carbon neutrality. Appl. Energy.

[29] Allwood J.M., Cullen J.M., Milford R.L. (2010). Options for Achieving a 50% Cut in Industrial Carbon Emissions by 2050. Environ. Sci. Technol..

[30] Chen L., Feng H., Xie Z. (2016). Generalized Thermodynamic Optimization for Iron and Steel Production Processes: Theoretical Exploration and Application Cases. Entropy.

[31] Chen Y., Yang S. (2021). Time-varying effect of international iron ore price on China’s inflation: A complete price chain with TVP-SVAR-SV model. Resour. Policy.

[32] Ma Y. (2021). Dynamic spillovers and dependencies between iron ore prices, industry bond yields, and steel prices. Resour. Policy.

[33] Faghih S.A.M., Kashani H. (2018). Forecasting Construction Material Prices Using Vector Error Correction Model. J. Constr. Eng. Manag..

[34] Jammazi R. (2012). Cross dynamics of oil-stock interactions: A redundant wavelet analysis. Energy.

[35] Fang S., Lu X., Li J., Qu L. (2018). Multifractal detrended cross-correlation analysis of carbon emission allowance and stock returns. Phys. A Stat. Mech. Its Appl..

[36] Guo S., Li H., An H., Sun Q., Hao X., Liu Y. (2019). Steel product prices transmission activities in the midstream industrial chain and global markets. Resour. Policy.

[37] Zhang D., Ji Q., Kutan A.M. (2019). Dynamic transmission mechanisms in global crude oil prices: Estimation and implications. Energy.

[38] An S., Gao X., An H., An F., Sun Q., Liu S. (2020). Windowed volatility spillover effects among crude oil prices. Energy.

[39] Zhang P., Ci B. (2020). Deep belief network for gold price forecasting. Resour. Policy.

[40] Sun Q.R., Gao X.Y., Wen S.B., Chen Z.H., Hao X.Q. (2018). The transmission of fluctuation among price indices based on Granger causality network. Phys. A Stat. Mech. Its Appl..

[41] Tiwari A.K., Oros C., Albulescu C.T. (2014). Revisiting the inflation-output gap relationship for France using a wavelet transform approach. Econ. Model..

[42] Shen J., Zheng B. (2009). Cross-correlation in financial dynamics. Eur. Lett..

[43] Jammazi R., Aloui C. (2015). Environment degradation, economic growth and energy consumption nexus: A wavelet-windowed cross correlation approach. Phys. A Stat. Mech. Its Appl..

[44] Ljung G.M., Box G.E.P. (1978). On a measure of lack of fit in time series models. Biometrika.

[45] Barrat A., Barthelemy M., Pastor-Satorras R., Vespignani A. (2004). The architecture of complex weighted networks. Proc. Natl. Acad. Sci. USA.

[46] Pei S., Makse H.A. (2013). Spreading dynamics in complex networks. J. Stat. Mech. Theory Exp..

[47] Freeman L.C. (1978). Centrality in social networks conceptual clarification. Soc. Netw..

